# Male Fertility and Reduction in Semen Parameters: A Single Tertiary-Care Center Experience

**DOI:** 10.1155/2012/649149

**Published:** 2012-01-26

**Authors:** D. Milardi, G. Grande, D. Sacchini, A. L. Astorri, G. Pompa, A. Giampietro, L. De Marinis, A. Pontecorvi, A. G. Spagnolo, R. Marana

**Affiliations:** ^1^Department of Obstetrics and Gynecology, International Scientific Institute “Paolo VI”, Catholic University of the Sacred Heart, Largo A. Gemelli 8, 00168 Rome, Italy; ^2^Unit of Endocrinology, Department of Clinical Medicine, Catholic University of the Sacred Heart, Largo A. Gemelli 8, 00168 Rome, Italy; ^3^Institute of Bioethics, Catholic University of the Sacred Heart, Largo A. Gemelli 8, 00168 Rome, Italy

## Abstract

*Background*. Infertility is both a clinical and a public problem, affecting the life of the couple, the healthcare services, and social environment. Standard semen analysis is the surrogate measure of male fertility in clinical practice. *Objective*. To provide information about the relationship between semen parameters and spontaneous conception. *Methods*. We evaluated retrospectively 453 pregnancies that occurred among 2935 infertile couples evaluated at an infertility clinic of a tertiary-care university hospital, between 2004 and 2009. *Results*. Normal semen analysis was present only in 158 patients; 295 subfertile patients showed alterations in at least one seminal parameter. A reduction in all seminal parameters was observed in 41 patients. Etiological causes of male infertility were identified in 314 patients. *Conclusion*. Our data highlights the possibility of a spontaneous conception with semen parameters below WHO reference values. Therefore, we support the importance of defining reference values on a population of fertile men. Finally, we analyzed the related ethical issues.

## 1. Introduction

Infertility is a common clinical problem. It affects 13% to 15% of the couples worldwide [1]. In addition, infertility is considered also a public problem. It does not only affect the couples' life, but it affects the healthcare services and social environment [2]. A male factor is solely responsible in about 20% of infertile couples and contributory in another 30–40% [3]. If a male infertility factor is present, it is almost always defined by the finding of an abnormal semen analysis. Clinicians usually rely on the results of semen analysis for the assessment of male fertility. Standard semen analysis is widely considered to be part of the routine assessment in evaluating male fertility and the surrogate measure of male fecundity in clinical practice [4, 5].

There is little consensus as to which parameter within a conventional semen analysis is the best predictor of male fertility. A correlation between sperm count and pregnancy has been reported in previous studies [6–8], showing evidence of a predictive value of sperm count. Therefore, WHO adopted the threshold of 20 × 10^6^/mL as reference for minimal value of sperm count [9]. Routine semen analysis also includes the evaluation of sperm morphology and motility; both of these parameters are considered to be related to male fertility [6–8, 10, 11].

However, since WHO reference values were adopted, it has become evident that a basic semen analysis is insufficient to determine the fertility status of the male since previous WHO reference values were made considering a “normal population” of healthy men, and not a population of men with proven fertility [12]. Due to this, serious concerns are raised about the possibility of conceiving with semen parameters below WHO cutoff values [7, 13, 14].

Additional sperm function tests, such as swelling and/or eosine test, do not provide further information to traditional semen parameters in the assessment of the fertility status of infertile male patients [15].

It is very difficult to identify a semen parameter and/or test capable in itself of predicting male fertility. This difficulty can lead to inaccurate diagnosis, inappropriate treatment, and unnecessary anxiety on the part of the patient [10]. Therefore, a correct interpretation of male subfertility could reduce the number of patients requiring assisted reproduction [16].

The role of the female partner must also be considered in the assessment of potential male fertility. Minor degrees of fertility impairment are not necessarily associated with couple infertility when present in only one partner, because the fertility status of the woman may compensate for the partner's subfertility [12].

We report a large retrospective study in a population of infertile couples who conceived after aetiological therapies. This study provides additional information regarding the relationship between semen parameters and spontaneous conception and regarding the indication for etiological treatments also in presence of severe reduction in semen parameters.

## 2. Materials and Methods

2935 infertile couples were evaluated at the infertility clinic of a tertiary-care university hospital [“A. Gemelli” University General Hospital, Rome (Italy)] between January 2004 and June 2009. All these couples were unable to conceive for at least 12 months. Each couple underwent evaluation by gynaecologists with expertise in infertility, supported by andrologists and endocrinologists. Physical examination, hormonal assessment of ovulatory function, screening for cervical and vaginal infections, transvaginal sonogram, evaluation of tubal patency, and genetic assessment (when indicated) were carried out in the female patients.

The male patients underwent physical examination. Genetic assessment, endocrine factor, nonendocrine testicular dysfunction, and accessory gland infections were also evaluated. Standard semen analysis was performed periodically (every two months) in trusted laboratories, according to WHO guidelines [9]. Etiological treatments were performed in men that showed identified causes of infertility, whereas empirical treatments (such as gonadotropins, antioxidants, carnitine, and aspartic acid) were performed in men with no identified causes of infertility.

Assisted reproductive techniques were not performed, according to the ethical guidelines of our institution. All couples expressed informed consent to this protocol.

453 couples obtained a spontaneous pregnancy. Clinical and laboratory data, including standard semen analysis, were collected from all couples. Semen parameters, obtained by semen analysis within 60 days before the cycle prior to conception, were evaluated in order to define seminal status at conception. Recent studies demonstrated that the intraindividual variation in seminal parameters has no clinical value and that there is no indication that intraindividual variation in semen parameters is more pronounced among men from infertile couples when compared with healthy controls [17]. Therefore, one ejaculate is a sufficient indicator of semen quality in a group of patients [18]. Semen analysis was classified according to WHO reference values. Patients were divided in different groups according to sperm count, motility, and morphology. Subsequently, we classified them in different groups according to the predictor value for pregnancies, as previously reported (oligospermia, asthenospermia, teratospermia, and oligoasthenoteratospermia) [1–5, 7]. Patients with a reduction in sperm count were also classified into 3 subgroups: severe (<5 mil/mL), moderate (5–15 mil/mL), and mild (15–20 mil/mL) oligospermia. For each group, we evaluated the mean ± SD of seminal parameters. In order to define the role of treatment in male subfertility, we also evaluated sperm parameters at admission and after the therapies, the last carried out before conception, in the group with oligoasthenoteratospermia (OAT).

## 3. Results

The average age of the male population was (mean ± SD) 36.05 ± 5.73 yrs, and of the female population was 33.62 ± 4.65 yrs, in the 453 couples that conceived. The mean duration of infertility was 29.08 ± 15.21 months. All the pregnancies occurred within 48 months after the first evaluation at our institute (Table 1). Known causes of male infertility were identified in 314 patients (69% of the pregnancies) (Table 2). Two causes of infertility were present simultaneously in 39 patients.

Amongst genital tract infections, *Ureaplasma urealyticum*, *Mycoplasma hominis*, and Gram-negative bacteria were the most recurrent (80% of the species identified). Varicocele was present in 115 patients (25% of patients), and 52 patients underwent varicocele repair (surgical varicocelectomy or scleroembolization).

**Figure 1:**
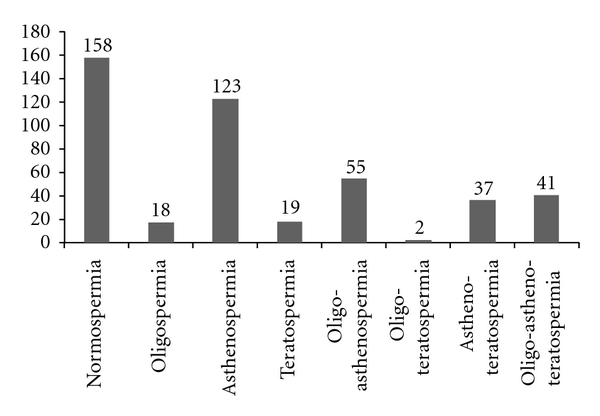
Classification of patients according to WHO reference parameters [9].

Normal semen analysis was present only in 35% of patients (158 pts), with sperm count 74.93 ± 40.16 × 10^6^/mL, linear motility 63.18 ± 12.51%, and normal morphology 53.74 ± 20.41%. 295 subfertile patients (65%) showed alterations in at least one seminal parameter, as reported in Figure 1.

A sperm count lower than WHO reference values, isolated or associated with motility or morphology alterations, was present in 116 patients (26% of the total pregnancies). Sperm count in this group was 9.09 ± 4.9 × 10^6^/mL. When stratified according to the grade of oligospermia, 13 patients presented mild oligospermia, 71 had moderate oligospermia, and 32 presented severe oligospermia (Table 3).

Isolated asthenospermia was the most frequent semen abnormality and was present in 123 patients (27% of the total pregnancies). Semen parameters in the asthenospermic group were sperm count 61.23 ± 34.79 × 10^6^/mL, linear motility 33.09 ± 11.25%, and normal morphology 55.51 ± 20.40%.

Isolated teratospermia was present in 19 patients (4% of the total pregnancies) and was associated with other seminal alterations in 80 patients (18% of the total pregnancies). Normal sperm morphology in isolated teratospermia was 22.29 ± 2.22%, and in associated teratospermia, it was 15.28 ± 10.40%.

41 pregnancies were obtained by men with OAT (9% of the total pregnancies). Sperm count in OAT group was 8.93 ± 5.12 × 10^6^/mL, linear motility was 24.36 ± 12.67%, and normal morphology was 13.00 ± 8.38%. In the OAT group, aethiological causes of male infertility were identified, isolated or associated, and treated in 31 patients. Male genital infections were appropriately treated in 28 patients, whereas hormonal disfunctions involving dysthyroidism, hypogonadism, and pituitary disfunctions were treated in 8 patients. Varicocele was treated in 3 patients.

When comparing seminal analysis before and after aethiological therapies in the OAT group, an increase in sperm count was observed in 3 patients, while an increase in linear motility was present in 15 patients. In 10 patients with idiopathic OAT, empirically different kinds of pharmacological treatments were tried: hMG/hCG, antioxidants, carnitine, and aspartic acid. An increase in linear motility was observed in 3 patients.

In the group of 52 patients who underwent varicocele repair, sperm count (mean ± SD) at admission was 9.06 ± 4.34 × 10^6^/mL, linear motility was 21.13 ± 7.12%, and normal morphology was 18.74 ± 3.93%. Semen analysis after surgery in the varicocele group showed a significative increase in sperm concentration (14.24 ± 3.12 × 10^6^/mL) and normal morphology (22.62 ± 3.05%) while a nonsignificative increase was observed in sperm motility (25.31 ± 8.27%).

## 4. Discussion

### 4.1. Altered Semen Parameters and Spontaneous Conception

Subfertility is a common condition affecting at least 15% of couples during their reproductive lives, and in half of these, a male factor is involved [1]. Recent evidence has questioned the clinical value of WHO criteria for basic semen analysis in the prediction of fecundity [8]. We report 453 pregnancies which occurred between January 2004 and June 2009 among 2935 infertile couples evaluated at our institute. In the infertile couples, we applied a standard protocol in terms of investigation and therapeutical management of couples affected by infertility, performed in both partners in a sequential and parallel way. This was achieved by a unified clinical management of the couple, which includes gynecologists, endocrinologists, and andrologists, with specific interest in the field of human reproduction.

Our data supports the possibility of spontaneous conception with semen parameters below the WHO reference values, as documented by the 65% of pregnancies that occurred in our center. A sperm count reduction was, in fact, observed in 116 patients (26% of the total pregnancies). 32 patients (27%) within the oligospermic group presented severe oligospermia showing a sperm count of 3.58 ± 1.54 × 10^6^/mL. An overlap between fertile and infertile patients in sperm concentration was previously reported [4, 6, 7]. Even though Bonde reported a predictive value for sperm concentration with increase up to 40 × 10^6^/mL [8], we present a group of severe oligospermic men in couples who conceived spontaneously. Astenospermia was the most frequent semen abnormality, both when evaluated as isolated or associated with other semen abnormalities. This high incidence is probably due to the high infection rate in our patients [19]. Previous studies on sperm motility as a predictor of infertility have proven contradictory [4, 6, 10, 11, 20]. This data seems to indicate that astenospermia may be a surmountable condition in infertile couples. A predictive value for normal morphology was previously reported [4, 6, 7, 10, 20] although only moderate predictive value for spontaneous pregnancy can be given. Conceptions were in fact reported by men with teratospermia [7]. Our data underscore the possibility of spontaneous conception also with reduction in normal sperm morphology. When we consider the percentage of normal morphology, it was near the lower end of reference values (22% for isolated teratospermia and 15% for associated teratospermia). Conception did not occur with normal morphology <5%. As a consequence, a conception with severe teratospermia might be improbable; therefore, we confirm that morphology could be considered the best predictive parameter for male fertility. We reported 41 pregnancies which occurred in men affected by oligoastenoteratospermia, even though Bonde and Ombelet described an increase in predictive value when altered seminal parameters were considered in combination with each other [7, 8]. These data highlight that seminal parameters, even when considered together as a whole, cannot exclude the possibility of a spontaneous conception. For these reasons, we sustain that semen parameters cannot lead to a clear-cut discrimination between fertile and infertile men.

### 4.2. WHO Reference Values for Seminal Parameters: What Is “Normality”?

Our data call into question the predictive value of WHO reference values as a diagnostic test, which are not always predictive of fertility. The WHO reference values [9] were drawn up considering a population of healthy men, disregarding the absolute minimal values of the semen parameter necessary in order for conception to occur. As a consequence, none of these parameters, considered alone or in combination, can be considered diagnostic of infertility [12]. In order to improve the clinical value of seminal parameters, WHO recently revised its laboratory manual for the examination and processing of human semen [21]. In this edition, the lower reference limit for seminal parameters is given at the 5th percentile in a population of men in couples who conceived within 12 months. The new WHO manual, considering as “normal population” the male of proven fertility, reduces previous reference values and is in agreement with our evidence of spontaneous conception by men with reduced seminal parameters. To date, there is no threshold in semen parameters which detects and predicts male fertility. New molecular insights into sperm properties which make it capable of fertilizing the egg are recently emerging. Increased knowledge of sperm proteome will allow us to have new predictors of molecular index in male fertility [22].

### 4.3. Etiological Treatments for Male Infertility: Waste of Time or Mandatory Practice?

According to medical literature, 30–40% of patients do not receive care based on available scientific evidence [23]. On top of this, an estimated 20–25% of provided health care is unnecessary [24]. In the case of subfertility, this could mean the use of unnecessary and expensive diagnostic tests and assisted reproductive techniques (ARTs) [25]. It is debated how long patients should be treated with medical and surgical therapies, strictly according to current clinical guidelines, when they present a significant range of uncertainty currently due to an incomplete understanding of etiology and physiopathology of a clinical condition.

Hughes et al. reported in a multicenter randomized trial significantly higher live birth rates per *in vitro* fertilization (IVF)/intracytoplasmatic sperm injection (ICSI) cycle when compared with no treatments for three months in women with Fallopian tube patency [26].

Another trial compared the effectiveness of immediate IVF with six-month delayed IVF in couples with all causes of infertility. Patients in the treatment group received up to four cycles of IVF treatment. Patients in the control group were permitted to have any form of fertility treatment other than IVF. Significant differences were observed in pregnancy rates per couple (17% with immediate IVF versus 8% with delayed IVF) and live birth rates per couple (12% with immediate IVF versus 5% with delayed IVF). No details of the fertility treatment received by the control group were presented [27]. A further randomized trial compared early IVF with late IVF (after six months) in couples with all causes of infertility. Patients in the treatment group received one cycle of IVF treatment. The control group received other fertility treatments during the six-month waiting period. Intention to treat analysis of all causes of infertility showed no significant differences in clinical pregnancy rates per couple (10% with immediate IVF versus 7% with delayed IVF), nor in live birth rates per couple (9% with immediate IVF versus 5% with delayed IVF).

According to these evidences, UK-NHS National Institute for Health and Clinical Excellence (NICE) Clinical Practise Guidelines (CPGs) for fertility assessment and treatment for people with fertility problems (2004) [28] reported that “the decision to recommend IVF treatment should take into consideration the likelihood of spontaneous pregnancy without treatment, in particular in cases where significant spontaneous pregnancy rates may be expected.” As a consequence, according to these CPGs, severe alterations in seminal plasma might be addressed immediately to IVF treatments.

In this paper, we report a conception in 453 couples with infertility, treated with only medical and surgical therapies; some of these presented severe alterations in seminal parameters. The mild improvement of seminal parameters in 18/31 patients affected by severe combined alterations in sperm count, motility, and morphology, via etiological treatments, suggests that no patient should be excluded from a complete diagnostic evaluation and etiological therapeutic opportunity, even in presence of severe alterations in seminal parameters. Therefore, the significant improvement in sperm concentration and morphology in selected patients who underwent varicocele repair underlines that varicocele repair may represent an effective option to the fertility.

To date, unique CPGs have not yet been developed regarding the indication and the timing for IVF techniques in relation to seminal parameters. These data should be therefore taken into consideration in evaluating current CPGs for couple infertility.

### 4.4. Clinical Epistemology and Ethics Issues Considerations

The present analysis raises two levels of considerations: the epistemology of the CPG in male infertility/subfertility treatment and the ethical issues related to this matter.

It is well known that the CPGs are a set of recommendations based on evidence as much as possible, able to change the behavior in specific clinical scenarios [29]. Particularly, the literature about male infertility shows that few clinical trials with pregnancy as the main outcome measure have been published. Consequently, WHO-CPG for male infertility are limited and in this area clinical practice is merely dominated by “authority-based” guidelines, even though WHO reference clinical parameters are the most widely used criteria to define a “male factor” [21].

Therefore, some points to be taken into consideration with regard to ethical issues concerned in male infertility treatment are as follows.

Firstly, the actual uncertainty of CPG on male infertility treatment (MIT): it is ethically correct to reach the infertile/subfertile patient/couple's best interest not in one-way only, that is, through the automatic shift towards ART, but also utilizing other convenient remedies, according to a clinical logic of “graduality” since it is sometimes possible to conceive a baby without recurring to ART.

Secondly, the dutifulness of an adequate counselling setting: In fact, the more recent literature [30–32] shows the need for psychological and ethical counselling for infertile/subfertile (naive and treated) couples. This data is also confirmed in ART legislation of many countries. For instance, in Italy the specific law no. 40/2004 supplies the couple with realistic information to face all the issues involved (benefits, risks, costs, clinical alternatives, and psycho-social/emotional aspects), ethical issues enclosed, because it is strictly linked to both patients' personal values/preferences and physicians' personal/professional values. At this condition, it would be possible to create and safeguard an adequate relationship, and we could contribute in increasing the autonomy of patient's mature and responsible choices, without leaving him/her alone.

Thirdly, the need for a further development of alternative remedies to solve/overcome fertility problems other than ART [32].

Finally, the ethical reflection on MIT—also pursued through an appropriate use of clinical ethics committees [33]—could contribute to give meaning to objective limitations of CPG, granting further value to the human subject. This is one more reason to not immediately address the choices towards ART, without first considering the relevant issues on distributive justice, fairness, and prioritization criteria of such expensive procedures [34–36].

## 5. Conclusions

The role of the endocrinologist/andrologist is primary and necessary for a complete diagnostic evaluation and etiological therapeutic opportunity, as reported in a large series of pregnancies achieved in a single tertiary-care center experience. We confirmed previous data about the impossibility for semen parameters to lead to a clear-cut discrimination between fertile and infertile men. As a consequence, no patient should be automatically shifted towards ART, even in presence of severe alterations in seminal parameters. ART techniques should be considered after a sufficient time of etiological therapies, also according to ethical criteria of distributive justice, fairness, and prioritization.

Therefore, unique CPGs have not yet been developed regarding the indication and the timing for IVF techniques in relation to seminal parameters. These data should be therefore taken into consideration in evaluating current CPG for couple infertility.

In this context, new molecular insights about sperm function in fertilizing the egg are recently emerging. Further studies are therefore needed in this topic in order to identify new molecular predictors of male fertility, which might solve this ancient dilemma about clinical decision in male infertility.

## Figures and Tables

**Table 1:** Time from the first evaluation to the conception.

No. of months	No. of patients	% of all the pregnancies
12	187	41.3
24	187	41.3
36	54	11.8
48	25	5.6

**Table 2:** Causes of male infertility.

Cause	No. of patients	% of all the pregnancies
Male tract infections	168	37
Varicocele	113	25
Hormonal disfunction (hypogonadism, dysthyroidism, pituitary disfunction)	72	16
Idiopathic infertility	100	22

**Table 3:** Stratification of the patients with reduction in sperm count according to the grade of oligospermia.

Grade of oligospermia (sperm count range)	Number of patients	Sperm count (mean ± SD)
Mild (16–20 × 10^6^/mL)	13	18,18 ± 0, 46 × 10^6^/mL
Moderate (5–15 × 10^6^/mL)	71	10,00 ± 2, 95 × 10^6^/mL
Severe (<5 × 10^6^/mL)	32	3,58 ± 1, 54 × 10^6^/mL
